# Acute polyneuropathy associated with Kambo poisoning: An unusual case report

**DOI:** 10.1016/j.toxrep.2026.102200

**Published:** 2026-01-05

**Authors:** Julio César Mantilla-Pardo, Juan David García-Valencia, Juan Pablo Fernández-Cubillos

**Affiliations:** a Servicio de Neurología, Fundación Valle del Lili, Cali, Colombia; b Facultad de Ciencias de Salud, Universidad Icesi, Cali, Colombia

**Keywords:** Acute peripheral neuropathy, Kambo, Intoxication, Immunoglobulin, Venom

## Abstract

Kambo is a natural secretion obtained from the Amazonian frog Phyllomedusa bicolor, traditionally used in ritualistic and alternative medicine practices for its purported purifying and immunostimulatory effects. Acute intoxication has been associated with neuropsychiatric manifestations, electrolyte disturbances, and systemic complications; however, involvement of the peripheral nervous system has not been previously confirmed by electrodiagnostic studies. We report the case of a 40-year-old man with no prior medical history who developed rapidly progressive quadriparesis and facial diparesis four days after subcutaneous self-application of Kambo venom. Cerebrospinal fluid analysis demonstrated albuminocytologic dissociation, and nerve conduction studies revealed an acute demyelinating motor polyneuropathy with conduction block and preserved sensory conduction. Due to clinical deterioration and risk of respiratory failure, the patient required intensive care management. He initially underwent five sessions of plasmapheresis with limited improvement, followed by intravenous immunoglobulin at a dose of 0.4 g/kg/day for five days, resulting in partial neurological recovery. At three-month follow-up, he persisted with residual motor deficits without sensory involvement. This case represents, to our knowledge, the first electrodiagnostically confirmed report of acute polyneuropathy associated with Kambo poisoning. Clinicians should be aware that Kambo intoxication may extend beyond central neuropsychiatric effects to involve the peripheral nervous system, and early recognition with consideration of immunomodulatory therapy may be warranted.

## Highlights

• First electrodiagnostic confirmation of neuropathy linked to Kambo intoxication.
• Acute demyelinating motor polyneuropathy developed after Kambo venom exposure.
• Possible immune-mediated mechanism responsive to immunotherapy.
• Partial recovery after plasmapheresis and intravenous immunoglobulin.
• Physicians should recognize Kambo as a potential cause of acute neuropathy.

## Graphical Abstract

**Figure:**
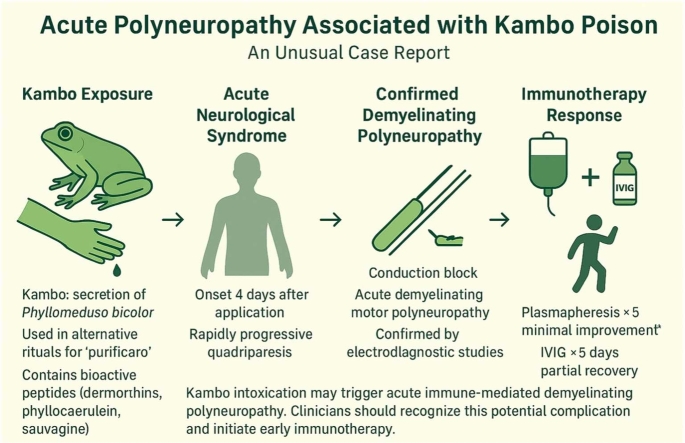

## 1 Introduction

Kambo is a natural substance derived from the glandular secretions of the Amazonian frog *Phyllomedusa bicolor*. Initially used by indigenous peoples of the Amazon as a traditional medicine, its use has spread to urban areas due to the belief that it possesses purifying and spiritual properties, as well as immune-boosting and physical performance–enhancing effects [1]. In Colombia, as well as the majority of countries, there are currently no public health regulations governing the self-application of *Kambo* venom.

Kambo intoxication typically presents as an acute, self-limited neuropsychiatric syndrome characterized by vomiting, hyponatremia, seizures, hallucinations, and psychosis [2]. Chronic neuropsychiatric sequelae have also been reported [3]. However, the effects of this toxin on the peripheral nervous system remain unclear. We report the first case of acute neuropathy associated with Kambo poisoning, confirmed by electrodiagnostic studies.

## 2 Case presentation

**Fig. 1:**
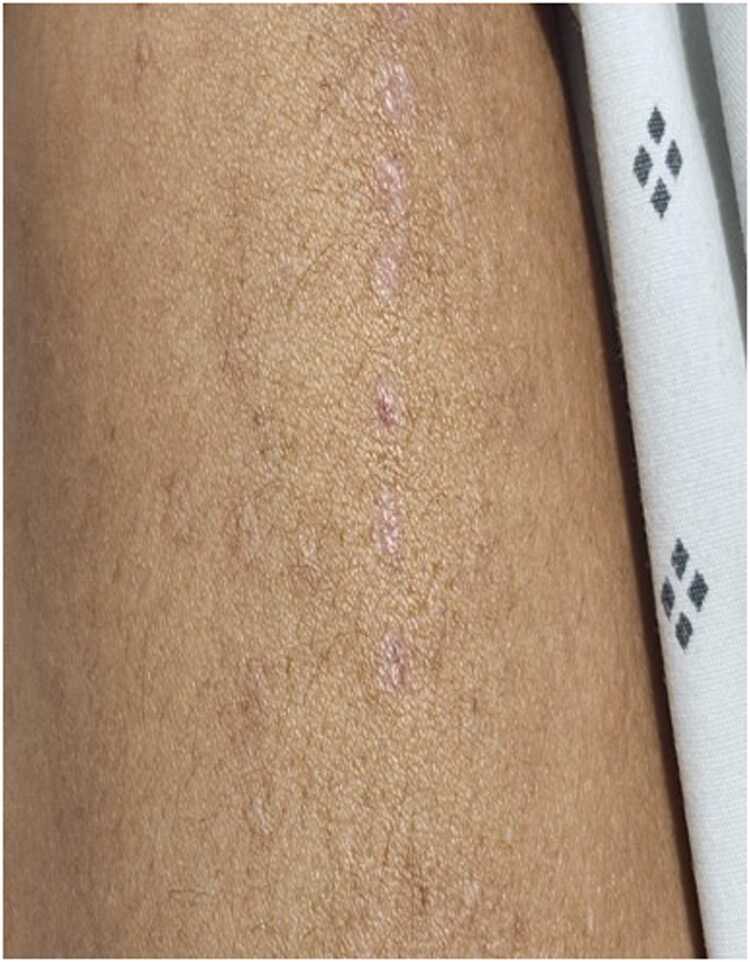
.

A 40-year-old Colombian male merchant, with no prior medical conditions was admitted to the emergency room. He reported a history of self-administered subcutaneous *Kambo* frog venom exposure every six months as part of spiritual rituals over the past eight years. The most recent exposure occurred on the right shoulder four days prior to symptom onset (Fig. 1). After self-application, he developed symmetrical progressive weakness in the upper limbs, which progressed over two days to involve all four limbs and neck flexor muscles. He denied any preceding gastrointestinal or respiratory illness or recent vaccinations. He reported an episode of drowsiness accompanied by nausea and vomiting. No bulbar symptoms were present.

Neurological examination revealed bilateral facial diparesis; Medical Research Council (MRC) muscle strength was 4/5 in neck flexors, 2/5 proximally and 1/5 distally in the upper limbs, 3/5 in hip flexors and knee extensors, and 1/5 in ankle dorsiflexors and plantar flexors. Deep tendon reflexes were absent in the upper limbs, with generalized hypopallesthesia and hypotonia.

**Fig. 2:**
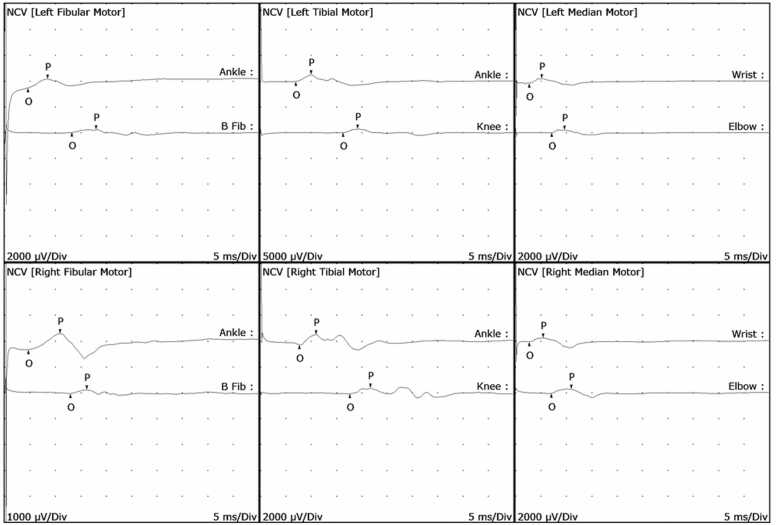
.

Initial laboratory studies including blood count test, renal and hepatic function, thyroid function, creatine phosphokinase (CPK), and electrolytes were within normal limits except for hyponatremia (130 mmol/L; reference range: 135–145 mmol/L). Rheumatological profile was negative. Cerebrospinal fluid (CSF) analysis showed elevated protein (88.9 mg/dL; reference range: 15–45 mg/dL) with normal cell count and negative microbiological studies. Human Immunodeficiency Virus (HIV) testing was negative. Sensory nerve conduction studies were normal. Motor conduction studies demonstrated acute demyelinating features affecting multiple nerves (Table 1), with evidence of conduction block (Fig. 2). Electromyography was normal.

**Table 1:** Nerve conduction study.

H Reflex Studies

NR: no recorded. Abnormal value are written in red in this table.

Due to the risk of respiratory failure, the patient was admitted to the intensive care unit (ICU) and underwent five sessions of plasmapheresis with minimal improvement, followed by intravenous immunoglobulin (IVIG) at a dose of 0.4 g/kg/day for five days. His weakness stabilized without respiratory compromise, and he was discharged with a home-based comprehensive rehabilitation plan, with global limb strength of 2/5 and neck flexor strength of 4/5.

**Fig. 3:**
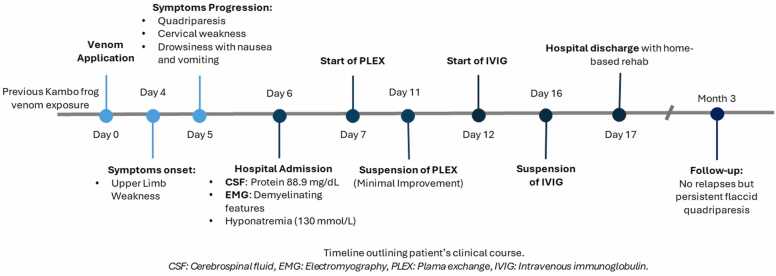
.

At a three-month follow-up, the patient continued to exhibit flaccid quadriparesis with distal predominance (MRC 2/5), without sensory deficits. Unfortunately, no new electrodiagnostic test was performed. Patient's clinical history is summarized in Fig. 3.

## 3 Discussion

The development of acute neuropathy following exposure to animal venoms is an uncommon but documented phenomenon. In a systematic review of acute peripheral neuropathy associated with animal envenomation [4], 54 cases were identified involving different animal venoms, suggesting that diverse animal toxins can trigger neuropathic syndromes through heterogeneous mechanisms.

In that review, the mean age was 34 years, and most patients were male. The majority presented with polyneuropathy, followed by focal neuropathy and myeloradiculoneuropathy, with a predominance of motor involvement. The onset of motor weakness generally occurred within five days since venom exposure. Reported causative animals included hymenopterans such as bees, wasps, hornets, and fire ants; other arthropods such as scorpions and spiders; aquatic animals such as jellyfish, venomous fish, and corals; and also snakes and lizards [4]. Notably, no cases associated with frog venom were reported.

Several pathophysiological mechanisms have been proposed for venom-induced neuropathies. These include direct neurotoxic effects of venom on nerves, early immune-mediated responses through molecular mimicry, vasculitic processes, toxic paranodopathy due to altered ion channel permeability, and delayed immune responses [4].

Kambo venom contains multiple bioactive peptides, including tachykinins, dermorphins, deltorphins, phyllocaerulein, and sauvagine, among others, which affect ion channels, peptide receptors, and the autonomic nervous system. Dermorphins and deltorphins are potent delta and mu opioid receptor agonists that may cause opioid-like toxicity. Tachykinins are implicated in pain mechanisms mediated via bradykinin B receptors. Phyllocaerulein stimulates the adrenal and pituitary glands, while sauvagine acts similarly to corticotropin-releasing hormone. Cerulein mimics cholecystokinin, leading to gastrointestinal effects such as vomiting, electrolyte loss, and hypertension [5], [6]. These molecules could theoretically act as haptens or induce tissue injury that triggers molecular mimicry and immune responses directed against peripheral nerve components. Therefore, immune-mediated mechanisms—either molecular mimicry or peripheral inflammatory activation—represent plausible pathophysiological hypotheses in this context.

Regarding management, Kambo intoxication is usually self-limited and primarily treated with supportive care and close monitoring, as no specific antidote exists. Intravenous rehydration is essential to correct fluid and electrolyte imbalances, and treatment should be tailored to the presenting symptoms [5]. Severe complications have been reported, including syndrome of inappropriate antidiuretic hormone secretion (SIADH), acute kidney injury, rhabdomyolysis, and esophageal rupture secondary to forceful vomiting, all of which require intensive medical management [3], [7], [8].

In the aforementioned case series, most patients recovered fully or nearly fully; however, a few achieved only partial recovery, and in rare cases, death was reported [4]. These patients generally received immunomodulatory therapy with corticosteroids, intravenous immunoglobulin (IVIG), or plasmapheresis, with IVIG being the most frequently administered [4], [9]. These data support the therapeutic approach used in our patient.

The differential diagnosis of acute polyneuropathy should include other etiologies such as immune-mediated neuropathies (e.g., Guillain–Barré syndrome), toxic neuropathies induced by heavy metals or drugs, infectious causes, and metabolic disorders such as acute thiamine deficiency, porphyria, or severe hyponatremic crises [10], [11].

Our patient showed a clear temporal association between Kambo application and symptom onset, without prior infection, vaccination, or other precipitating factors. He exhibited typical features of acute Kambo intoxication—altered consciousness, nausea, vomiting, and hyponatremia—followed by the development of motor symptoms. His partial response to plasmapheresis and IVIG supports a possible acute immune-mediated mechanism responsive to immunotherapy, while the incomplete recovery may reflect secondary axonal damage, delayed treatment initiation, or mixed mechanisms (direct toxicity and immune response).

To the authors’ knowledge, this is the first electrodiagnostically confirmed case of acute neuropathy associated with Kambo frog venom. A previous case reported in Mexico described a patient who developed sensory disturbances after Kambo application—hypoesthesia of the left index finger and generalized hypopallesthesia—suggesting focal sensory neuropathy; however, no electrodiagnostic confirmation was obtained [12].

Given the reports of complications and fatalities, it is essential to warn the public about the potential adverse effects of Kambo use. The neurotoxic effects are not limited to acute neuropsychiatric presentations but may also involve the peripheral nervous system and lead to chronic neuropsychiatric sequelae. Public health policies should be strengthened to regulate the use of these peptides, as has occurred in Brazil, where regulatory agencies have prohibited the promotion and use of this alternative practice [13].

## 4 Conclusion

The use of Kambo frog venom is a common practice within alternative medicine. Physicians should be aware of its potential complications. Although most cases present as an acute, self-limited neuropsychiatric syndrome, Kambo intoxication may manifest atypically with involvement of not only the central but also the peripheral nervous system. We report the first electrodiagnostically confirmed case of acute polyneuropathy associated with Kambo venom intoxication.

## Ethical considerations

The patient provided written informed consent to have this case published. The study involving human subjects was approved by the institution’s ethical board (Comité de Ética en Investigación Biomédica IRB, Fundación Valle del Lili, Cali, Colombia). Institutional ethics board approval was received on December 2025 with the reference number 229–2025. All procedures were performed in compliance with relevant laws and institutional guidelines.

## Funding

This research did not receive any specific grant from funding agencies in the public, commercial or non-for-profit sectors.

## CRediT authorship contribution statement

**Juan David García-Valencia:** Writing – review & editing, Validation, Software, Resources, Project administration. **Julio César Mantilla-Pardo:** Writing – original draft, Validation, Supervision, Investigation, Formal analysis, Conceptualization. **Juan Pablo Fernández-Cubillos:** Writing – review & editing, Visualization, Project administration, Methodology, Investigation, Conceptualization.

## Declaration of Competing Interest

The authors declare that they have no known competing financial interests or personal relationships that could have appeared to influence the work reported in this paper.

## Data Availability

No data was used for the research described in the article.
